# Phillyrin Alleviates Memory Impairment in D‐Galactose‐Induced Aged Mice by Suppressing Hippocampal Neuroinflammation via the TLR4/NF‐κB Signaling Pathway

**DOI:** 10.1002/fsn3.72241

**Published:** 2026-08-07

**Authors:** Xuemin Li, Liru Wang, Chenyang Li, Junfeng Huo, Shuqin Li, Linxiu Bian, Ying Zhang, Yongfei Bai, Jie Yao, Xinyuan Hao

**Affiliations:** ^1^ School of Public Health Shanxi Medical University Taiyuan Shanxi PR China; ^2^ Center for Disease Control and Prevention in Shanxi Province Taiyuan Shanxi PR China; ^3^ The Second Clinical Medical College of Shanxi Medical University Taiyuan PR China; ^4^ Taiyuan Iron and Steel (Group) co., Ltd. General Hospital Taiyuan Shanxi PR China

**Keywords:** aged model, dietary bioactive compound, neuroinflammation, oxidative stress, phillyrin, TLR4/NF‐κB signaling pathway

## Abstract

Phillyrin (PHI), a major dietary lignan derived from *Forsythia suspensa* , is a neuroprotective bioactive compound. This study investigated the protective effects of PHI against D‐galactose (D‐gal)‐induced memory decline in mice and explored the underlying mechanisms, focusing on neuroinflammation mediated by Toll‐like receptor 4 (TLR4) and nuclear factor kappa B (NF‐κB). In vivo, mice were assigned to control, D‐gal, and PHI treatment groups. Behavioral performance, hippocampal histopathology, oxidative stress, inflammatory markers, and related protein expression were evaluated. In vitro, HT22 cells exposed to D‐gal were treated with PHI, TAK‐242, or both, and analyzed for TLR4/NF‐κB proteins by Western blotting (WB). PHI attenuated cognitive impairment, reduced neuroinflammation, and preserved synaptic structure. Moreover, PHI downregulated TLR4, p‐IκBα, and p‐NF‐κB expression both in hippocampal tissue and HT22 cells, consistent with the effects of the TLR4 inhibitor TAK‐242. These findings suggest that PHI exerts neuroprotective and anti‐inflammatory effects via TLR4/NF‐κB modulation, highlighting its potential as a dietary bioactive compound to support cognitive health during aging.

## Graphical abstract

Phillyrin (PHI), a dietary bioactive lignan isolated from *Forsythia suspensa* , alleviates D‐galactose‐induced cognitive impairment by suppressing the TLR4/NF‐κB signaling pathway. PHI inhibits TLR4‐mediated activation of NF‐κB, thereby reducing neuroinflammation and oxidative stress, preserving neuronal structure and synaptic plasticity, and ultimately improving learning and memory function. These findings highlight the potential of PHI as a dietary bioactive compound to support cognitive health during aging.

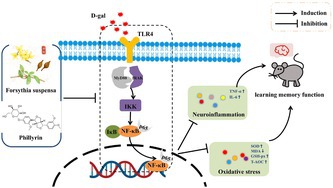

## 1 Introduction

Aging is a natural and irreversible biological phenomenon characterized by the gradual decline of physiological functions at the cellular, tissue, and systemic levels, which is closely linked to cellular stress responses, impaired energy metabolism, and stem cell exhaustion (Moqri et al. 2023). According to projections by the World Health Organization (WHO) and the United Nations, the global number of people aged 60 and older is projected to reach nearly 2.1 billion by 2050, accounting for about 22% of the total population (Ganesan et al. 2019). Aging is commonly associated with progressive impairments in brain function and cognitive performance. Due to its sensitivity to aging, the brain gradually develops multilevel degenerative changes, laying the groundwork for neurodegenerative diseases in older adults (Mattson et al. 2002). The Global Burden of Disease Study projects that the number of people living with dementia will rise to 152.8 million globally by 2050 (GBD 2019 Dementia Forecasting Collaborators 2022). This alarming trend poses serious challenges to public health systems and highlights the pressing need to develop more effective preventive and therapeutic measures.

Chronic administration of excessive D‐gal can induce the accumulation of reactive oxygen species (ROS) and trigger oxidative stress, thereby accelerating the aging process. Previous studies have shown that the D‐gal‐induced aging model can effectively mimic several pathological features of natural aging, including enhanced oxidative stress, neuroinflammation, and functional impairment in the central nervous system (Saleh et al. 2019; El‐Mesery et al. 2024). Therefore, this model has been widely used in studies investigating aging and age‐related cognitive dysfunction.

Oxidative stress and neuroinflammation are major factors contributing to synaptic damage and cognitive impairment during brain aging (Chung et al. 2009; Coyle and Puttfarcken 1993). As the aging process progresses, the endogenous antioxidant defense system becomes progressively impaired, as evidenced by decreased activities of superoxide dismutase (SOD), glutathione peroxidase (GSH‐Px), and total antioxidant capacity (T‐AOC), along with elevated malondialdehyde (MDA) levels, indicating redox imbalance and an increased risk of neuronal injury (Teleanu et al. 2022). Meanwhile, the decline in learning memory function, attributable to various factors, exhibits a strong correlation with the inflammatory response. During this process, Toll‐like receptor 4 (TLR4) recognizes and binds specific ligands, leading to the phosphorylation and degradation of IκBα, which allows the NF‐κB p65 subunit to translocate into the nucleus and initiate the transcription of proinflammatory cytokines, including tumor necrosis factor‐α (TNF‐α) and interleukin‐6 (IL‐6), thereby aggravating inflammation and contributing to neuronal damage (Feng et al. 2016). The excessive release of these cytokines further promotes neuronal damage, consequently worsening learning and memory impairments. Multiple animal studies have confirmed that the occurrence and progression of age‐related cognitive impairment are closely associated with aberrant activation of the TLR4/NF‐κB signaling pathway (Chen, Guo, et al. 2024). Targeting the TLR4/NF‐κB pathway has emerged as a promising approach to delaying brain aging and mitigating memory decline.

*Forsythia suspensa* (Thunb.) Vahl, a medicinal plant of the Oleaceae family, is native to China and widely distributed in East Asia (Wang et al. 2018). Forsythiae Fructus (FF), the dried fruit of *F. suspensa* , is traditionally used for its heat‐clearing and detoxifying properties, especially in treating fever, inflammation, and sore throat (Cho et al. 2010). In addition to its medicinal uses, *F. suspensa* is also consumed as a dietary ingredient. Its leaves can be brewed into tea, which has been safely consumed for centuries and is traditionally believed to relieve internal heat and inflammation. This edible use supports the recognition of phillyrin as a dietary bioactive compound with potential health‐promoting effects during aging. Phillyrin (PHI, C_27_H_34_O_11_), a lignan glycoside, is one of the major bioactive constituents of FF and is used as a phytochemical marker for quality control of this herb (Bai et al. 2015; Fang et al. 2013). PHI has attracted considerable attention because of its multiple biological activities, including antibacterial (Zhou et al. 2019), anti‐inflammatory (Jiang et al. 2020; Zhang et al. 2020), antioxidant (Li et al. 2023), antiviral (Qu et al. 2016), and anti‐cancer effects (Wang et al. 2019). Moreover, recent studies have reported that PHI mitigates neuronal damage in Parkinson's disease models, potentially by enhancing mitochondrial function and autophagy (Qi et al. 2024). In addition, PHI has demonstrated strong anti‐inflammatory properties against severe lipopolysaccharide (LPS)‐induced neutrophil inflammation by downregulating the MyD88‐dependent pathway and inhibiting the activation of MyD88, IκBα, and NF‐κB (Chen, Li, et al. 2024).

Despite these findings, the neuroprotective potential of PHI in brain aging remains largely unexplored. Therefore, the present study aims to evaluate the protective effect of PHI on memory performance in D‐gal‐induced aged models in vivo and in vitro, and to explore whether this effect is mediated via suppression of the TLR4/NF‐κB signaling axis. To further confirm the regulatory effect of PHI on the TLR4/NF‐κB signaling pathway, the expression of its key proteins was investigated in D‐gal‐induced HT22 cells.

## 2 Materials and Methods

### 2.1 Animals and Treatments

**FIGURE 1:**
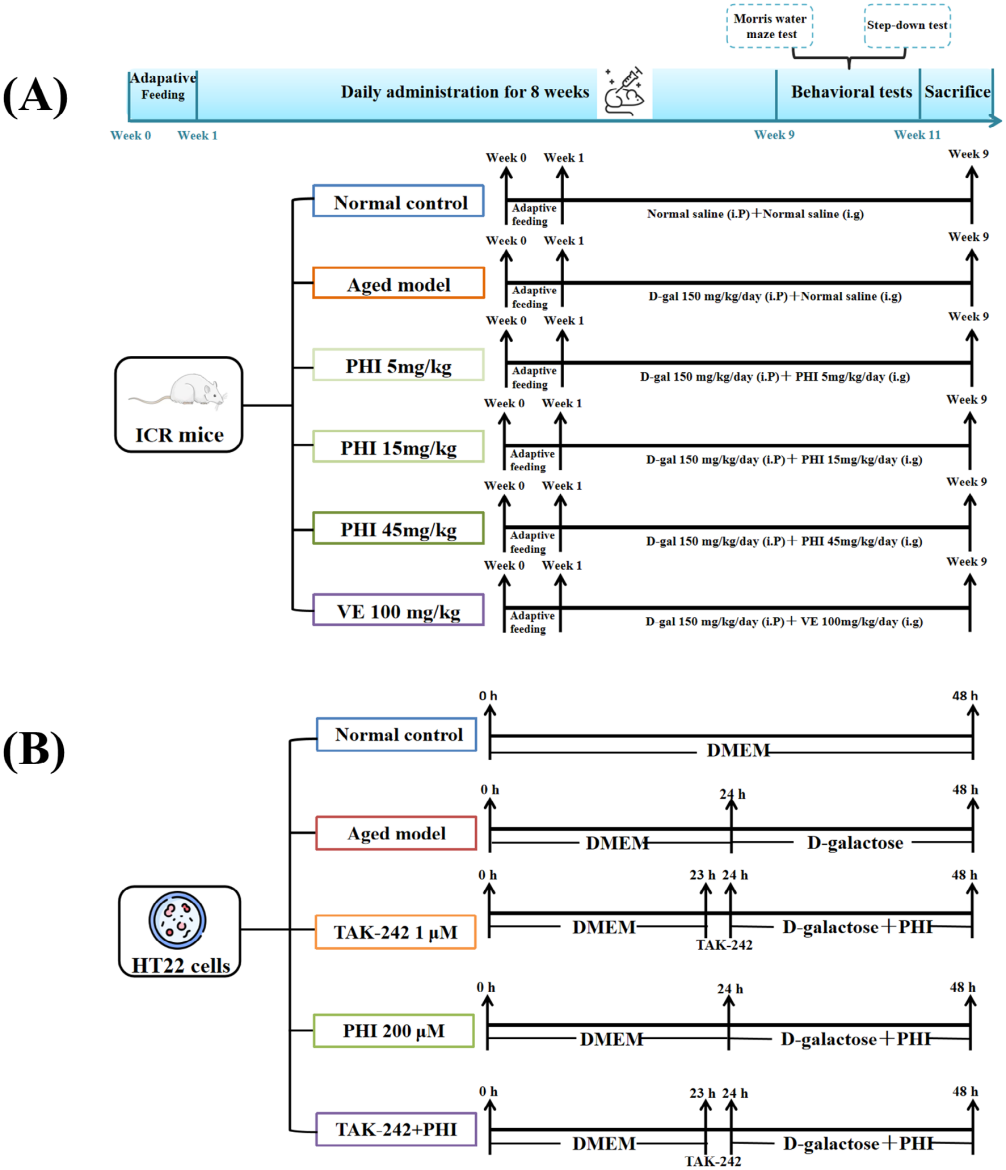
The treatment of mice and cells in each group. (A) The treatment of mice in each group. (B) The treatment of cells in each group.

Female Institute of Cancer Research (ICR) mice (6–8 weeks, 25–30 g) were purchased from Shanxi Medical University (License No. SCXK (Jin) 2019‐0004). All mice were housed in the Animal Laboratory of the Shanxi Provincial Center for Disease Control and Prevention (License No. SYXK (Jin) 2020‐0005). Animals were housed under controlled conditions (20°C–26°C, 40%–70% relative humidity) with a 12 h light/dark cycle and had free access to standard chow and water. All animal experimental procedures were approved by the Ethics Committee of the Center for Disease Control and Prevention in Shanxi Province (Approval ID: 20230710), and were conducted in accordance with the ARRIVE guidelines and the National Institutes of Health Guide for the Care and Use of Laboratory Animals. Following a 1‐week acclimation period, mice were randomly divided into six groups (*n* = 10 per group). Except for the Normal control group, the other five groups were intraperitoneally injected with D‐gal (150 mg/kg/day, dissolved in 0.9% normal saline) for 8 weeks. At the same time, mice in the PHI treatment groups received PHI (5, 15, or 45 mg/kg/day, dissolved in 1% methylcellulose) by oral gavage, while mice in the VE group received VE (100 mg/kg/day, dissolved in corn oil) by oral gavage. VE was used as a positive control because of its confirmed antioxidant and anti‐aging effects (Sun et al. 2018). The doses of PHI and VE used in this study were selected based on previous studies (Yan et al. 2015; Omidi et al. 2019). PHI and VE administration were initiated simultaneously with D‐gal treatment and continued throughout the experimental period; therefore, this study was designed to evaluate the preventive effect of PHI against D‐gal‐induced aging (Figure 1A). Phillyrin (PHI, commercially labeled as Forsythin), a major bioactive lignan glycoside of Forsythiae Fructus, was used in this study to represent the active principle of the traditional herbal medicine. PHI (HPLC purity ≥ 98%, Batch No. C15993816) and D‐galactose (D‐gal) were purchased from Sichuan Weikeqi Biological Technology Co. Ltd. (Chengdu, China) and Beijing Solarbio Science and Technology Co. Ltd. (Beijing, China), respectively. The purity of PHI was confirmed according to the supplier's Certificate of Analysis (CoA). HPLC chromatogram is provided in Figure S1. After behavioral testing, the mice were euthanized, and brain and final body weights were recorded to calculate the brain index (mg/g).

### 2.2 Cell Culture and Treatment

HT22 cells, a mouse hippocampal neuronal cell line, were purchased from Procell Life Science & Technology Co. Ltd. (Wuhan, China). Cells were seeded into cell culture flasks at a density of 1–5 × 10^5^/mL and cultured in DMEM (Gibco, Shanghai, China) supplemented with 10% fetal bovine serum (Cellmax, Beijing, China) and 1% penicillin/streptomycin (Meilunbio, Dalian, China) at 37°C in 5% CO_2_ for 24 h. After reaching 80%–90% confluence, they were treated with D‐gal and PHI of different concentrations for 24 h. Subsequently, cell viability was measured by cell counting kit‐8 (CCK‐8; Meilunbio, Dalian, China), and the cell survival rate was calculated. To clarify the underlying mechanism, HT22 cells were pretreated with TAK‐242 (TLR4 inhibitor) for 1 h before interventions were added (Cui et al. 2020). Cells were divided into the Normal control, Aged model, TAK‐242 1 μM, PHI 200 μM, and TAK‐242 + PHI groups. The treatment of cells in each group were shown in (Figure 1B).

### 2.3 Morris Water Maze Test (MWMT)

A circular pool (22°C ± 1°C) was used and filled with opaque water. The experiment included acquisition trials and a spatial probe trial. During acquisition, mice were trained for 5 consecutive days (4 trials per day) to locate the submerged platform. For the probe test, the platform was removed, and the mice were released into the pool from the southwest quadrant. Swimming trajectory, number of platform passes, and duration spent in the target quadrant were recorded and analyzed using the SMART v3.0 behavioral analysis system.

### 2.4 Step‐Down Test

Mice were placed on an insulated platform in a chamber with an electrified grid floor and allowed to accommodate for 3 min without stimulation. During the 5‐min training session, a foot shock (36 V, 1.5 mA) was delivered upon stepping down. After 24 h, the mice underwent a second probe test identical in procedure. The step‐down latency and error times were automatically recorded using a YLS‐3 TB step‐down recording apparatus (Jinan Yiyan Technology Development Co. Ltd., Jinan, China).

### 2.5 Hematoxylin and Eosin (HE) Staining

Mouse brains were immersed in 4% paraformaldehyde solution at 4°C for 48 h to ensure adequate fixation, followed by dehydration, clearing, and embedding in paraffin blocks. The paraffin‐embedded hippocampal tissues were subsequently sectioned and stained with HE.

### 2.6 Transmission Electron Microscope (TEM)

Hippocampal tissues were first immersed in 3% glutaraldehyde solution for primary fixation and subsequently treated with 1% osmium tetroxide for post‐fixation at 4°C. After washing, samples were dehydrated in graded ethanol (30%–100%) for 15 min each, followed by acetone treatment for 20 min. Ultrathin sections with a thickness of approximately 60–70 nm were prepared and carefully mounted onto copper grids. The sections were then stained in the dark with 2% uranyl acetate for 20 min, followed by counterstaining with lead citrate for an additional 5 min. Images were obtained using a JEM‐1400FLASH TEM (JEOL, Tokyo, Japan).

### 2.7 Biochemical Assays

The activities of SOD and GSH‐Px, the levels of T‐AOC and MDA in hippocampus were measured according to the kit instructions (Jiancheng Institute of Biological Engineering, Nanjing). Commercial Enzyme‐linked immunosorbent assay (ELISA) kits were used to measure levels of TNF‐α and IL‐6 in hippocampus following the manufacturer's instructions (Jiangsu Meimian Industrial Co. Ltd).

### 2.8 Western Blot (WB)

The hippocampal samples were gently washed twice with cold PBS for 5 min each. The BCA Protein Assay Kit (Boster, Wuhan, China) was used to determine the total protein concentration. Proteins were separated using 8%–10% SDS‐PAGE and transferred to polyvinylidene fluoride (PVDF) membranes. The membranes were blocked with 5% fat‐free milk at 37°C for 1 h and incubated overnight at 4°C with primary antibodies. The primary antibodies used in this test included: anti‐P53 (1:1000, ABclonal, China), anti‐TLR4 (1:1000, GeneTex, USA), anti‐IRAK, anti‐p‐IκBα, anti‐NF‐κB p65, and anti‐p‐NF‐κB p65 (1:1000, Affinity Biosciences, USA), anti‐β‐actin (1:2000, Servicebio, China). β‐actin was used as the internal loading control for all WB analyses. The following HRP‐conjugated secondary antibodies were used after three washes in TBST at room temperature: rabbit anti‐rat IgG (1:3000; Boster, China), goat anti‐rabbit IgG (1:3000; Boster, China). Protein bands were visualized using an ECL detection kit (Abbkine, Wuhan, China). Image J was used to analyze the protein band intensities.

### 2.9 Statistical Analysis

Statistical analysis was performed using SPSS 26.0 software and GraphPad Prism 8.0. Behavioral data were expressed as mean ± SEM, other data were presented as mean ± SD. Comparisons between individual groups were conducted using one‐way analysis of variance (ANOVA). The LSD or Dunnett T3 method was used for post hoc analysis. *p* < 0.05 was considered statistically significant.

## 3 Results

### 3.1 Effects of PHI on Body Weight and Brain Index of Mice

Following 8 weeks of D‐gal treatment, weight gain of mice was significantly reduced in the Aged model group compared to the Normal control group (*p* < 0.01). Treatment with 15 mg/kg and 45 mg/kg PHI markedly improved weight gain compared to the Aged model group (*p* < 0.01, *p* < 0.05, respectively). A similar effect was observed in VE treatment group (*p* < 0.05).

The brain index in the Aged model group was markedly lower than that in the Normal control group (*p* < 0.01). However, this reduction was significantly attenuated by 45 mg/kg PHI and VE treatment (*p* < 0.05 for all) (Table 1).

**TABLE 1:** Effects of PHI on body weight and brain index.

Groups	Weight gain (g)	Brain Index (mg/g)
Normal control	12.32 ± 1.17	15.14 ± 1.84
Aged model	8.45 ± 1.30**	13.08 ± 0.81**
PHI 5 mg/kg	9.82 ± 1.87	13.24 ± 0.94^$^
PHI 15 mg/kg	10.39 ± 1.94^##^	13.73 ± 0.57
PHI 45 mg/kg	10.19 ± 1.94^#^	14.37 ± 0.39^#^
VE 100 mg/kg	10.27 ± 0.75^#^	14.65 ± 1.78^#^

*Note:* Data were shown as mean ± SD (*n* = 8). **p < 0.01 vs. Normal control group; ^#^ *p* < 0.05, ^##^ *p* < 0.01 vs. Aged model group; ^$^ *p* < 0.05 vs. 100 mg/kg VE group.

### 3.2 PHI Ameliorated Memory Impairment and Reduced the Expression of P53 in the Hippocampus of the Aged Mice

**FIGURE 2:**
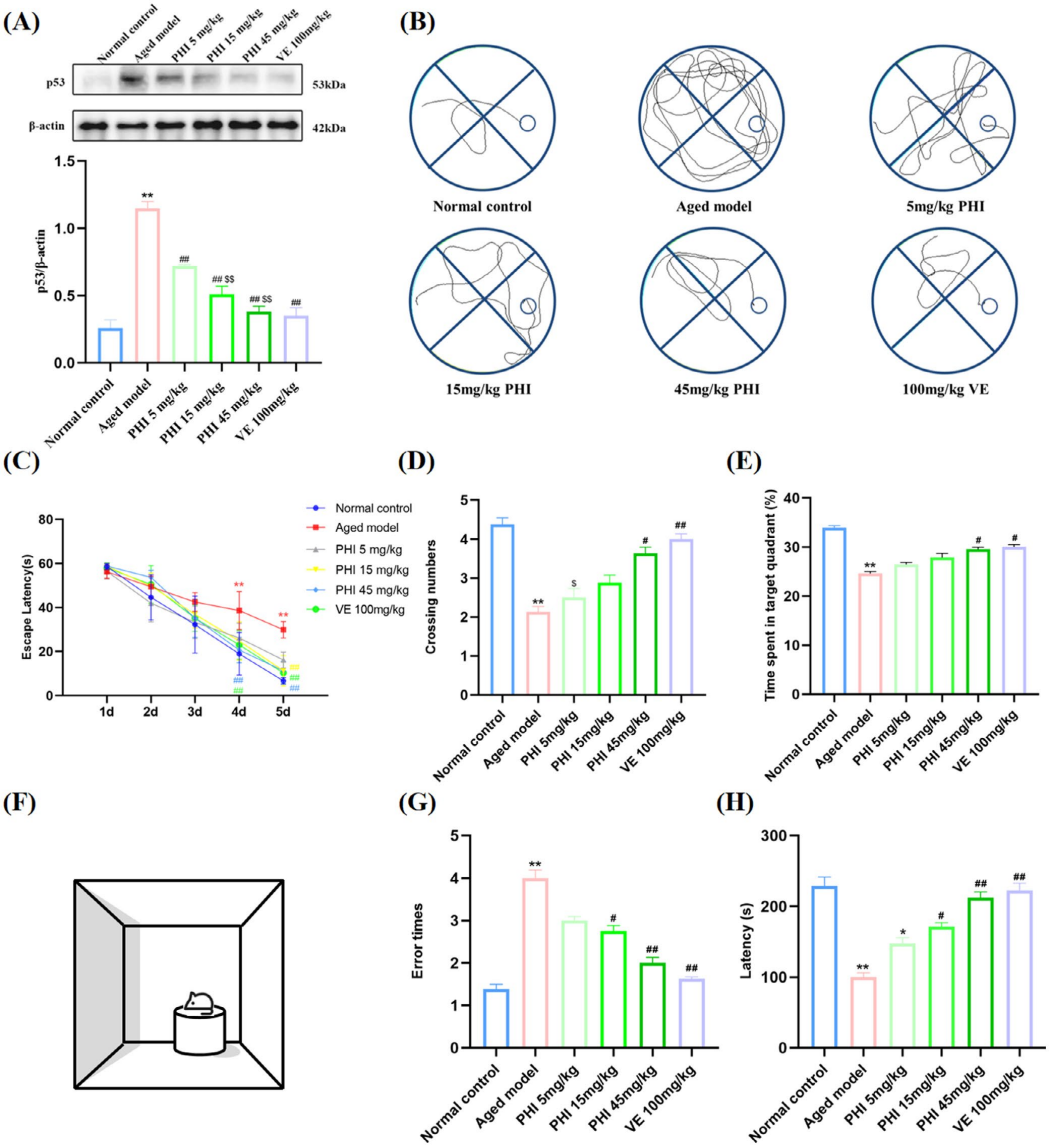
Effects of PHI on hippocampal P53 expression and memory in D‐gal‐induced aged mice. (A) PHI reduced the expression of P53 in the hippocampus of the D‐gal‐induced aged mice. The band was a representative of the protein (*n* = 3, mean ± SD). (B) Representative swimming trajectories during the spatial probe test. (C) Escape latency of positioning navigation trials. (D) Number of platform crossings during the spatial probe test. (E) The percentage of time spent in the target quadrant. (F) Schematic diagram of the step‐down. (G, H) Latency and error times in the step‐down. Data in B–H are presented as mean ± SEM (*n* = 8). *p < 0.05, **p < 0.01 vs. Normal control group; ^#^ *p* < 0.05, ^##^ *p* < 0.01 vs. Aged model group; ^$^ *p* < 0.05, ^$$^ *p* < 0.01 vs. 100 mg/kg VE group.

P53 levels were elevated in the Aged model group relative to the Normal control group (*p* < 0.01). The expression of P53 was down‐regulated in the 5, 15, and 45 mg/kg PHI group compared with the Aged model group (*p* < 0.01 for all). Similar reductions occurred in the VE treatment group (*p* < 0.01) (Figure 2A).

To evaluate whether PHI treatment could alleviate memory impairment in Aged model mice, the MWMT was conducted. Trajectory analysis showed that mice in the Aged model group exhibited disorganized swimming paths, whereas mice treated with PHI or VE displayed more directed swimming paths (Figure 2B). During the 5‐day training period, escape latency gradually declined in all groups as training progressed. However, on the 4th and 5th days, the escape latency of the Aged model group was significantly longer than that of the Normal control group (*p* < 0.01 for all). Compared with the Aged model group, mice treated with PHI at 15 mg/kg showed a significantly reduced escape latency on Day 5 (*p* < 0.01), whereas PHI at 45 mg/kg and VE at 100 mg/kg significantly reduced escape latency on both Days 4 and 5 (*p* < 0.01 for all) (Figure 2C). In the spatial probe test, the number of platform crossings was significantly decreased in the Aged model group compared with the Normal control group (*p* < 0.01), while treatment with 45 mg/kg PHI (*p* < 0.05) and VE (*p* < 0.01) significantly increased the number of crossings (Figure 2D). Likewise, the percentage of time spent in the target quadrant was significantly reduced in the Aged model group (*p* < 0.01), but was effectively reversed by treatment with 45 mg/kg PHI and VE treatment group (*p* < 0.05 for all) (Figure 2E).

During the step‐down test, the Aged model mice demonstrated a significantly shorter latency to receive electric shocks (*p* < 0.01) and increased number of errors compared to the Normal control group (*p* < 0.01). Treatment with 15 and 45 mg/kg PHI led to longer avoidance latency and fewer errors compared with the Aged model group (*p* < 0.05, *p* < 0.01). Similar results were found in the VE 100 mg/kg group (*p* < 0.01) (Figure 2G,H).

### 3.3 Effects of PHI on Hippocampus Histology in Mice

**FIGURE 3:**
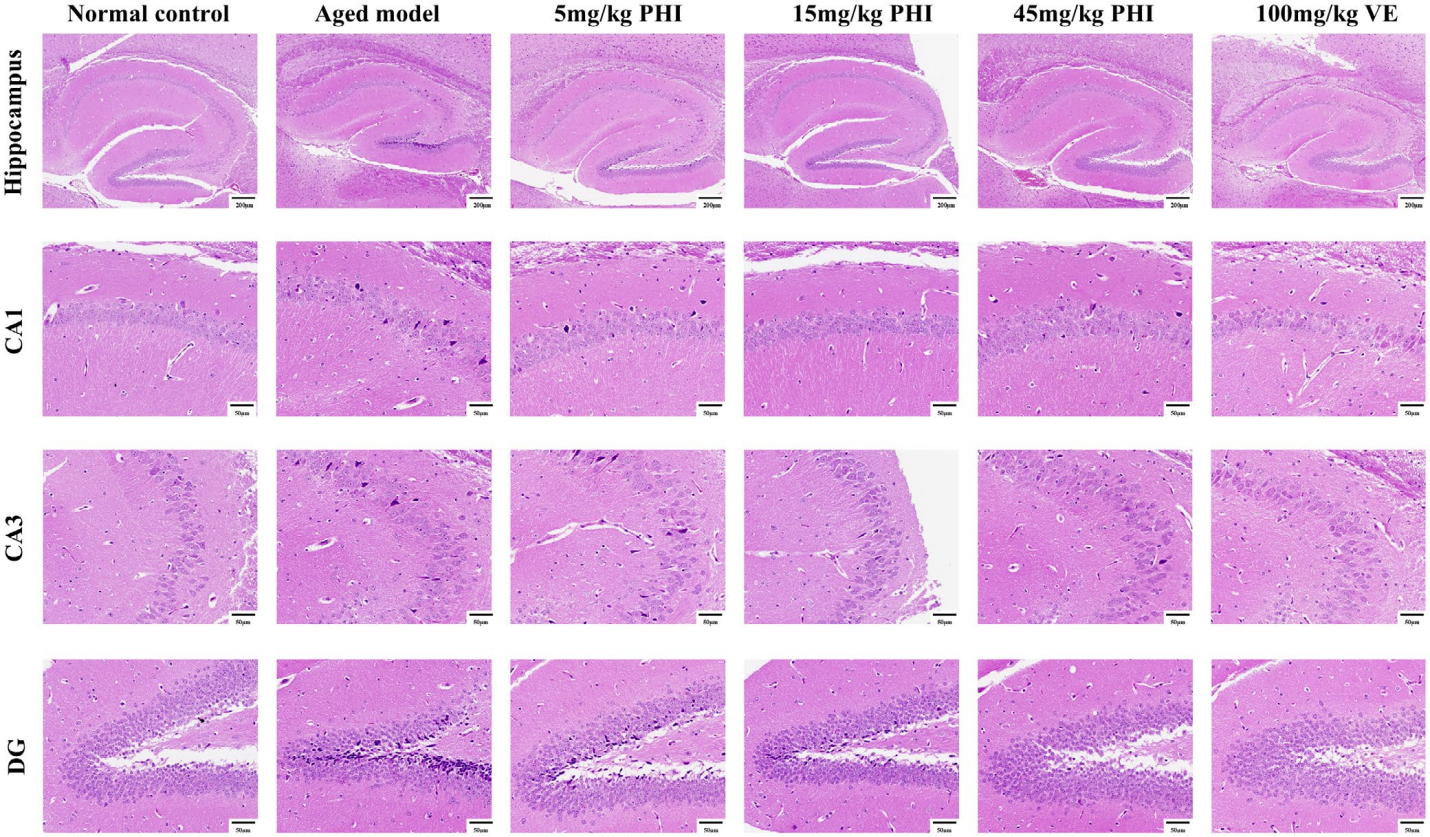
Effects of PHI on morphological alterations in the hippocampal subregions of aged mice (original magnification 400×, *n* = 3).

In the Normal control group, HE staining revealed that hippocampal nerve cells were neatly arranged with normal morphology, full cytoplasm, and clearly defined nuclei. In the Aged model group, cell arrangement appeared disordered and density decreased, while morphological abnormalities and neuronal degeneration were also evident. In the PHI and VE treatment groups, neuronal morphology and structure appeared more intact, and the arrangement was relatively regular (Figure 3).

### 3.4 PHI Ameliorated the Synaptic Structure of the Hippocampus in Aged Mice

**FIGURE 4:**
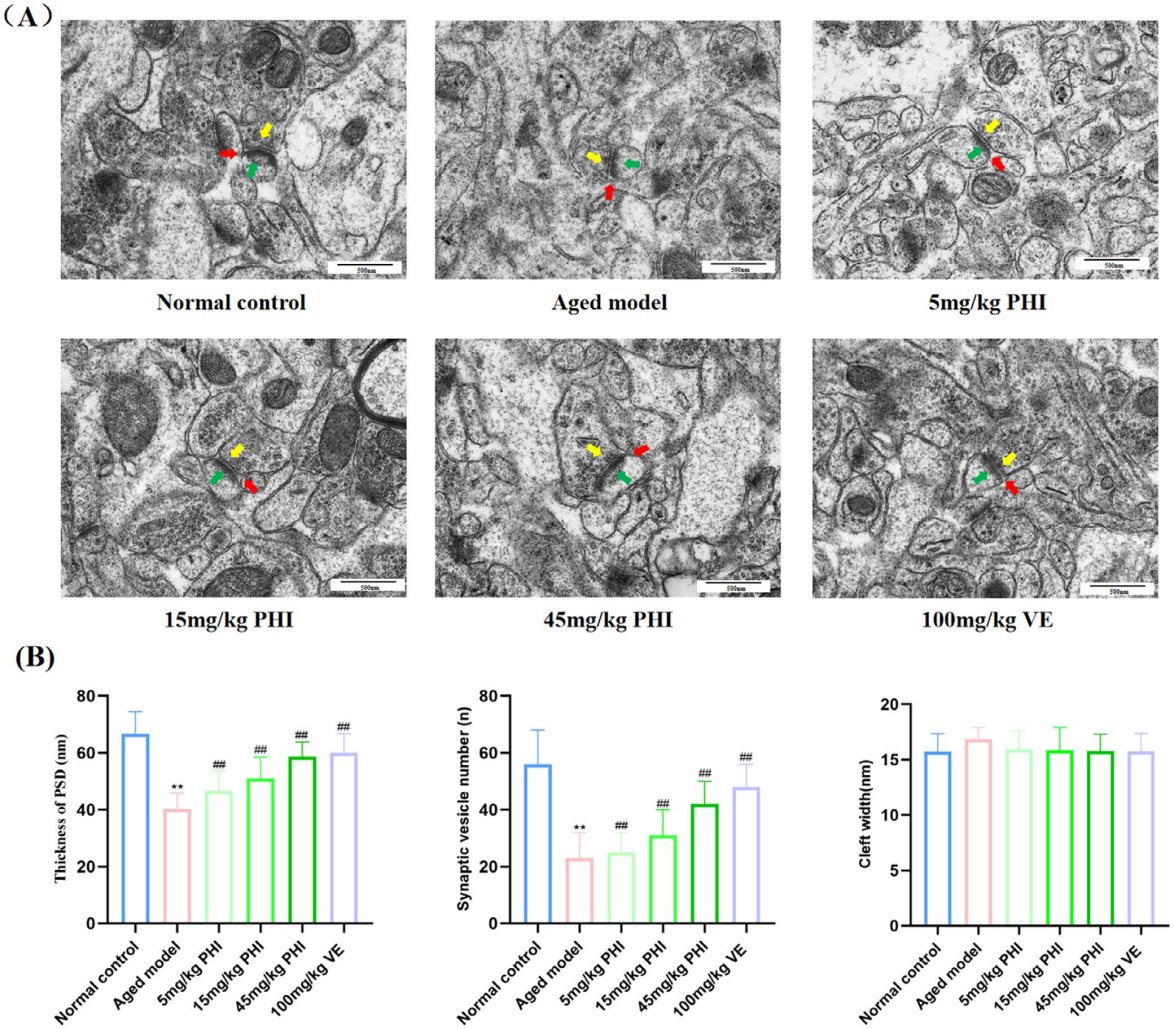
PHI ameliorated synaptic ultrastructural damage in the hippocampus of D‐gal‐induced mice. (A) TEM images showed PHI administration alleviated the destruction of the synaptic ultrastructure of the hippocampus. Representative TEM images of the presynaptic membrane (green arrow), synaptic vesicles (yellow arrow), and synaptic cleft (red arrow) in the hippocampus (*n* = 3; scale bar = 500 nm). (B) Quantitative comparison of synaptic interface parameters in the hippocampal CA1 region. Data were presented as mean ± SD. **p < 0.01 vs. normal control group; ^##^ *p* < 0.01 vs. aged model group.

TEM analysis revealed clear changes in the synaptic ultrastructure within the hippocampal CA1 region (Figure 4A). Compared with the Normal control group, the Aged model group showed a reduced number of synaptic vesicles and decreased thickness of the postsynaptic density (PSD) (*p* < 0.01). Treatment with 5, 15, and 45 mg/kg PHI improved synaptic structure, as evidenced by increased vesicle number and thickened PSD (*p* < 0.01 for all). A similar improvement was also observed in the VE 100 mg/kg group (*p* < 0.01) (Figure 4B).

### 3.5 Effects of PHI on SOD and GSH‐Px Activities and T‐AOC and MDA Levels in the Hippocampus

**FIGURE 5:**
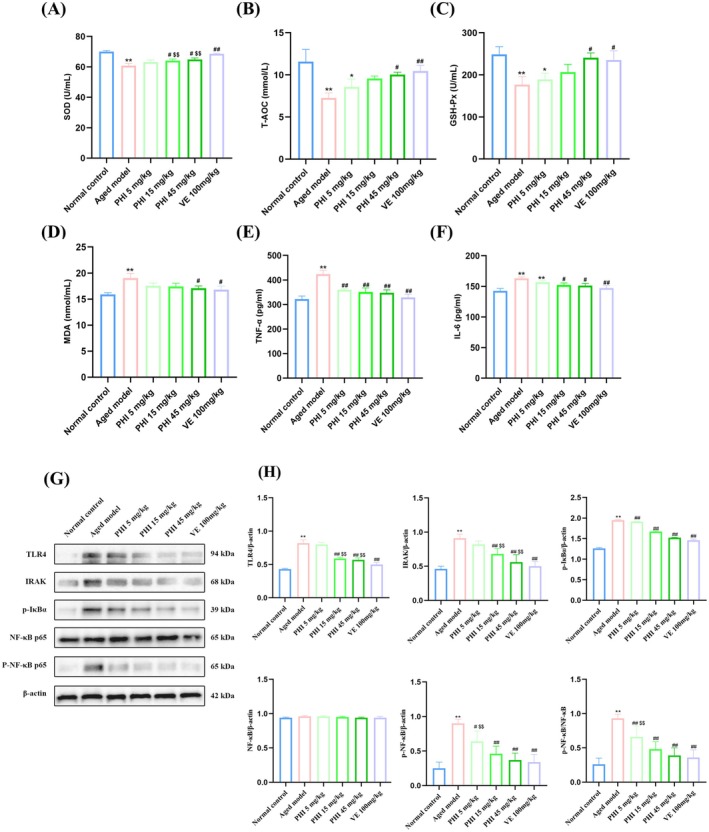
PHI enhanced antioxidant capacity, reduced inflammation, and modulated TLR4/NF‐κB signaling in the hippocampus of D‐gal‐induced aged mice. (A–D) SOD and GSH‐Px activities, and T‐AOC and MDA levels in the hippocampus. (E–F) ELISA results showed the levels of TNF‐α and IL‐6 in the hippocampus. (G–H) Effects of PHI on the related protein expressions of TLR4/NF‐κB signaling pathway in hippocampus. The bands were representatives of several blots. Data were presented as mean ± SD (*n* = 6 for biochemical and ELISA assays; *n* = 3 for WB). *p < 0.05, **p < 0.01 vs. Normal control group; #*p* < 0.05, ^##^ *p* < 0.01 vs. Aged model group; ^$^ *p* < 0.05, ^$$^ *p* < 0.01 vs. 100 mg/kg VE group.

To evaluate the effect of PHI on oxidative stress in D‐gal‐induced aged mice, the activities of SOD and GSH‐Px and the levels of T‐AOC and MDA in the hippocampus were measured. In the Aged model group, the activities of SOD, GSH‐Px, and the level of T‐AOC were markedly reduced, whereas the level of MDA was significantly elevated compared to the Normal control group (*p* < 0.01 for all). Compared with the Aged model group, treatment with PHI at 15 and 45 mg/kg significantly increased SOD activity, while 45 mg/kg PHI also significantly increased GSH‐Px and T‐AOC activities and decreased MDA levels (*p* < 0.05 or *p* < 0.01). Similar effects were observed in the VE 100 mg/kg group. In addition, SOD activity in the VE 100 mg/kg group was significantly higher than that in the PHI 15 mg/kg and PHI 45 mg/kg groups (*p* < 0.01 for all) (Figure 5A–D).

### 3.6 Effects of PHI on TNF‐α and IL‐6 Levels in the Hippocampus of Mice

ELISA analysis indicated that the hippocampal levels of TNF‐α and IL‐6 were significantly elevated in the Aged model group compared to the Normal control group (*p* < 0.01). Compared with the Aged model group, the level of TNF‐α was significantly down‐regulated in all PHI‐treated groups (*p* < 0.01 for all), and the level of IL‐6 was significantly reduced in 15 mg/kg and 45 mg/kg PHI groups (*p* < 0.05 for all). A similar reduction in TNF‐α and IL‐6 levels was also observed in the VE 100 mg/kg group (*p* < 0.01 for all) (Figure 5E,F).

### 3.7 Effects of PHI on the Protein Expressions in the TLR4/NF‐κB Signaling Pathway

Accumulating evidence suggests that the TLR4/NF‐κB signaling pathway is critically involved in neuroinflammatory processes. As shown in Figure 5G, in comparison with the Normal control group, the hippocampal expression of TLR4, IRAK, p‐IκBα, and p‐NF‐κB p65 proteins was significantly higher in the Aged model group (*p* < 0.01 for all), whereas no significant difference was observed in total NF‐κB p65 expression (*p* > 0.05). Compared with the Aged model group, PHI at 5 mg/kg significantly reduced the expression of p‐IκBα and p‐NF‐κB p65, whereas PHI at 15 and 45 mg/kg significantly decreased the expression levels of TLR4, IRAK, p‐IκBα, and p‐NF‐κB p65 (*p* < 0.05 or *p* < 0.01). Similar inhibitory effects were observed in the VE‐treated group (*p* < 0.01) (Figure 5H).

### 3.8 PHI Reduced D‐Gal‐Induced Cellular Senescence in HT22 Cells

**FIGURE 6:**
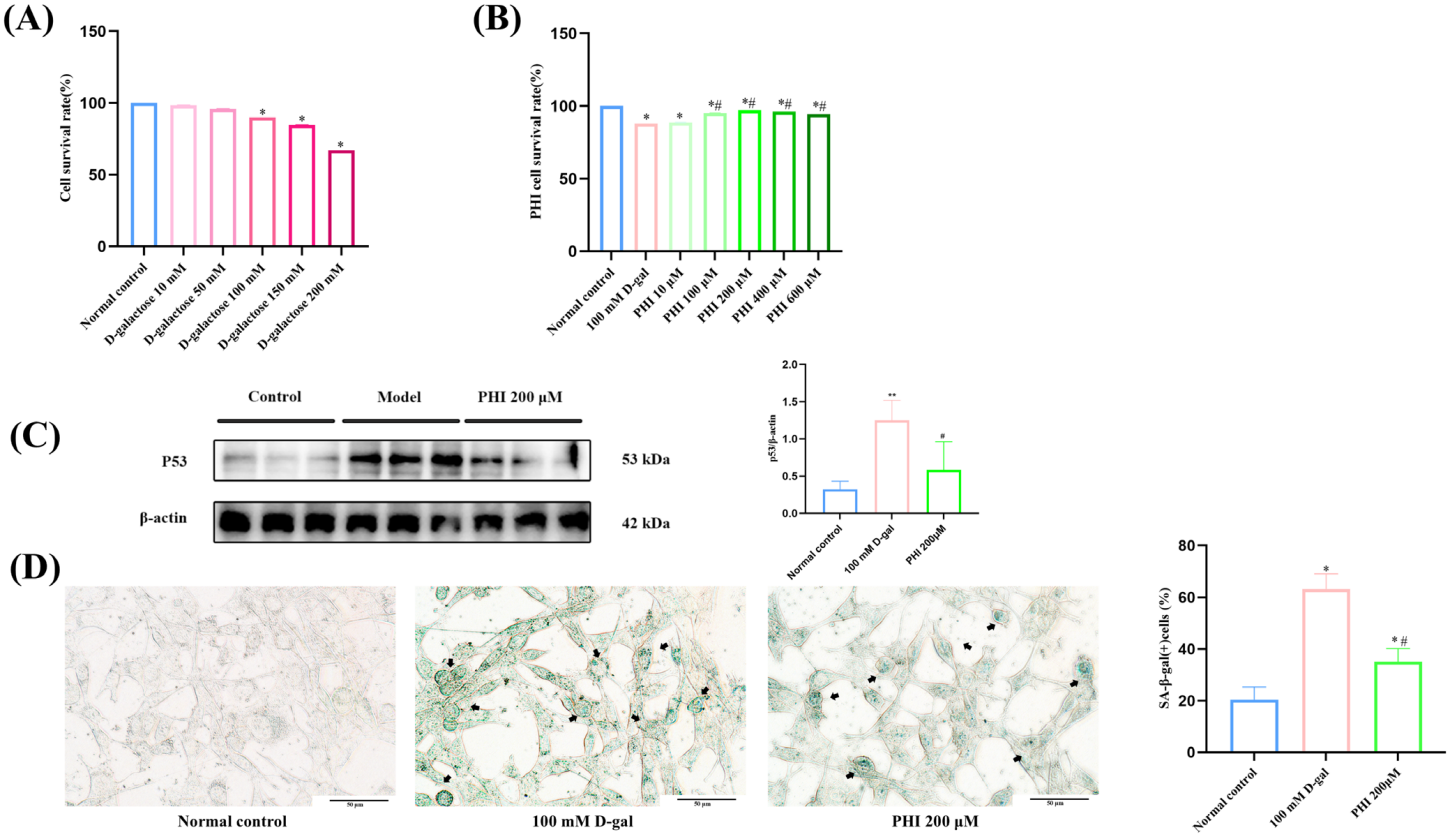
PHI alleviated D‐gal‐induced cellular senescence in HT22 cells. (A, B) The effects of different concentrations of D‐gal and PHI on cell viability (*n* = 3). (C) Representative WB images and quantification of P53 protein expression. Data were expressed as mean ± SD (*n* = 3). (D) Typical SA‐*β*‐gal staining images of HT22 cells and the percentage of SA‐*β*‐gal‐positive cells (400×, *n* = 3, scale bar = 50 μm). *p < 0.05, **p < 0.01 vs. normal control group; ^#^ *p* < 0.05 vs. 100 mM D‐gal group.

Based on the findings in vivo, we further validated the key effect of the NF‐κB pathway in HT22 cells. First, CCK8 results showed that D‐gal 100, 150, and 200 mM significantly reduced cell viability. Among them, cells treated with 100 mM D‐gal maintained a viability greater than 80%, and this concentration was suitable for subsequent experiments (Figure 6A). The survival rate of cells treated with 200 μM PHI was significantly higher than that of the 100 mM D‐gal group (Figure 6B). Senescence‐associated beta‐galactosidase (SA‐*β*‐gal) staining and P53 expression were further assessed to verify the successful establishment of the cellular 100 mM D‐gal. The results showed that the percentage of SA‐*β*‐gal‐positive cells and the expression levels of P53 in the 100 mM D‐gal group were significantly more than those in the Normal control group (*p* < 0.05 for all) (Figure 6C,D). PHI 200 μM effectively reduced these indexes, indicating that PHI can alleviate cellular senescence in D‐gal‐induced HT22 cells (*p* < 0.05 for all).

### 3.9 TLR4 Inhibitor TAK‐242 Promoted the Anti‐Senescence Effect of PHI in the Aged Model Cells

**FIGURE 7:**
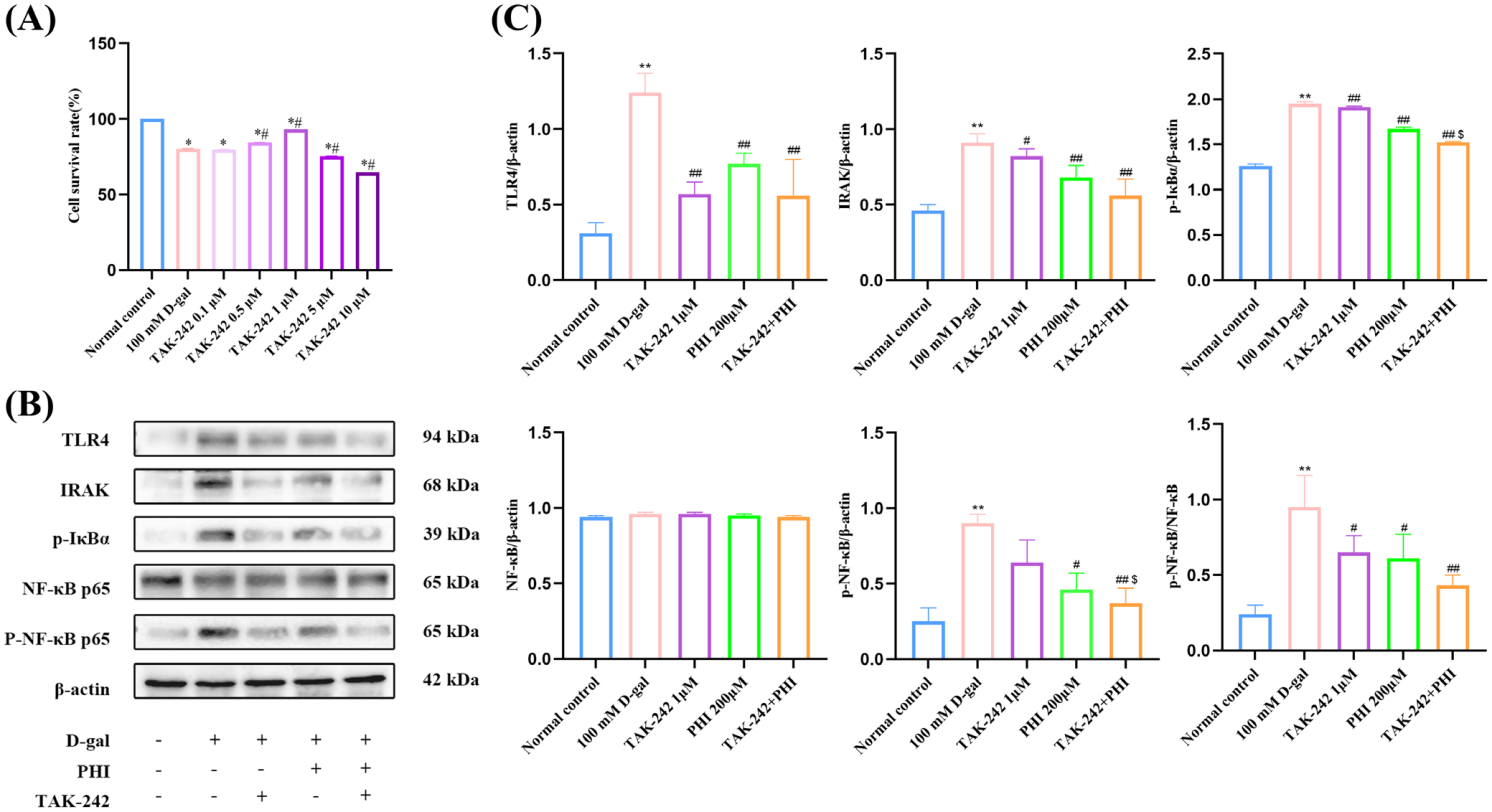
PHI inhibited D‐gal‐induced activation of the TLR4/NF‐κB signaling pathway in HT22 cells. (A) Determination of the optimal concentration of TAK‐242 (*n* = 3). (B, C) Effects of PHI on the expressions of proteins related to the TLR4/NF‐κB signaling pathway in HT22 cells. The bands were representatives of several blots. All data were presented as the mean ± SD (*n* = 3). *p < 0.05, **p < 0.01 vs. normal control group; #*p* < 0.05, ^##^ *p* < 0.01 vs. 100 mM D‐gal group; $*p* < 0.05 vs. PHI 200 μM group.

To further investigate whether PHI exerts its anti‐aging effect via the TLR4/NF‐κB signaling pathway, HT22 cells were pretreated with TAK‐242. Cells pre‐treated with 1 μM TAK‐242 showed the highest viability and were therefore selected for subsequent experiments (Figure 7A). We next examined the expression of key signaling proteins to confirm the effective inhibition of the TLR4/NF‐κB pathway. The expression of TLR4, IRAK, p‐IκBα, and p‐NF‐κB p65 proteins was notably higher in the 100 mM D‐gal group than in the Normal control group (*p* < 0.01 for all), whereas no significant difference was observed in total NF‐κB p65 expression (*p* > 0.05). Treatment with 200 μM PHI significantly reduced the expression levels of TLR4, IRAK, p‐IκBα, and p‐NF‐κB p65 compared with the 100 mM D‐gal group (*p* < 0.05 or *p* < 0.01). In addition, pretreatment with TAK‐242 decreased the expression levels of TLR4, IRAK, and p‐IκBα (*p* < 0.05 or *p* < 0.01). The TAK‐242 + PHI group also exhibited significantly reduced expression levels of TLR4, IRAK, p‐IκBα, and p‐NF‐κB p65 compared with the 100 mM D‐gal group (*p* < 0.01 for all). Furthermore, compared with the PHI 200 μM group, the expression levels of p‐IκBα and p‐NF‐κB p65 were further reduced in the TAK‐242 + PHI group (*p* < 0.05) (Figure 7B,C).

## 4 Discussion

In this study, we demonstrated that PHI alleviates memory impairment by inhibiting the TLR4/NF‐κB signaling pathway in D‐gal‐induced aged mice model. D‐gal is a commonly used and effective agent for inducing aging‐like phenotypes, as prolonged administration mimics several features of natural aging (Xia et al. 2022). According to the Alzheimer's Association, women account for two‐thirds of Alzheimer's disease cases in the United States (Alzheimer's Association 2021). In our study, the aged model was established in female ICR mice via daily intraperitoneal injection of D‐gal for 8 consecutive weeks, considering the greater susceptibility of females to age‐related cognitive decline. P53 is a key cell cycle regulatory protein that can trigger the aging process by inducing growth arrest (Rufini et al. 2013). Compared with the Normal control group, mice in the Aged model group showed significantly increased P53 protein expression (Figure 2A) and elevated hippocampal MDA levels, while the activities of SOD and GSH‐Px as well as the level of T‐AOC in the hippocampus were significantly decreased (Figure 5A–D). These results collectively indicate that the aged model was successfully established (Cui et al. 2006; Song et al. 1999).

PHI treatment significantly improved spatial reference memory in the MWMT and passive avoidance memory in the step‐down test in D‐gal‐induced aged mice (Figure 2B–H). Similar cognitive improvement was also reported in traumatic brain injury (TBI) models, where PHI mitigated neuronal damage and preserved blood–brain barrier (BBB) integrity, possibly by suppressing microglia‐mediated neuroinflammation through the PPARγ/NF‐κB signaling pathway (Jiang et al. 2021). The hippocampus is the critical region responsible for learning and memory. Earlier research indicates that D‐gal‐induced aging leads to neuronal degeneration, synaptic disruption, and morphological abnormalities in the hippocampus, ultimately contributing to cognitive decline (Bettio et al. 2017). Our study also demonstrated that D‐gal administration caused synaptic damage in the hippocampus of mice. Notably, PHI treatment alleviated this structural impairment and preserved neuronal morphology and synaptic integrity, suggesting its potential neuroprotective effects in the aging brain (Figures 3 and 4).

D‐gal induces significant oxidative stress, reflected by decreased SOD and GSH‐Px activities and elevated MDA levels. This redox imbalance may activate the TLR4/NF‐κB pathway, leading to inflammation and neuronal damage (Zhang and Ghosh 2001). Ma et al. demonstrated that PHI alleviates inflammatory responses and cartilage deterioration in osteoarthritis by reducing chondrocyte inflammation through blocking the NF‐κB pathway and decreasing the expression of proinflammatory mediators such as TNF‐α, COX‐2, IL‐6, and iNOS (Ma et al. 2024). We examined key proteins in the TLR4/NF‐κB signaling cascade. Compared to the Normal control group, elevated expression of TLR4, IRAK, p‐IκBα, and p‐NF‐κB p65 in the hippocampus was observed in the Aged model mice (Figure 5G,H). Consistently, ELISA results revealed increased levels of TNF‐α and IL‐6 in the hippocampus. PHI treatment significantly reduced the expression of these proteins and pro‐inflammatory cytokines (Figure 5E,F).

To further verify whether PHI exerts neuroprotective effects by inhibiting the NF‐κB signaling pathway, a 100 mM D‐gal‐induced aging model was established in HT22 cells in vitro. TAK‐242, a specific TLR4 inhibitor, significantly suppressed D‐gal‐induced activation of the TLR4/NF‐κB signaling pathway, as evidenced by decreased expression levels of TLR4, IRAK and p‐IκBα. Furthermore, compared with PHI treatment alone, the combination of TAK‐242 and PHI further reduced the expression levels of p‐IκBα and p‐NF‐κB p65 (Figure 7B,C). Our findings are supported by other studies. For example, Du et al. provided evidence that PHI reduced oxidative stress and attenuated apoptosis in H_2_O_2_‐induced ARPE‐19 cells by inhibiting the NF‐κB and p38 MAPK signaling pathways (Du et al. 2020). Taken together, these results indicate that PHI improves memory impairment and mitigates aging‐related neuronal damage by suppressing neuroinflammation through the TLR4/NF‐κB signaling pathway, providing experimental evidence for its potential as an anti‐aging therapeutic candidate.

## 5 Conclusion

In summary, PHI significantly improves memory in D‐gal‐induced aged mice, which may be attributed to the inhibition of the TLR4/NF‐κB signaling pathway, reduction of neuroinflammation, and protection of neuronal structure and synaptic plasticity. Overall, these findings suggest that PHI may serve as a dietary bioactive compound with potential to promote cognitive health and support healthy aging.

Nevertheless, this study has some limitations. Although the TLR4/NF‐κB signaling pathway was evaluated using total protein lysates, subcellular fractionation was not performed to verify the nuclear translocation of NF‐κB p65. The molecular mechanisms of PHI have not yet been fully validated at the gene transcription or multi‐omics levels, and its direct interaction with TLR4‐related receptor complexes remains unclear. Future studies integrating transcriptomics and proteomics are needed to further identify its molecular targets and support its development as a potential anti‐neurodegenerative intervention.

## Author Contributions

**Xuemin Li:** conceptualization, methodology, resources, project administration. **Chenyang Li:** writing – review and editing. **Linxiu Bian:** investigation. **Liru Wang:** writing – original draft, writing – review and editing. **Junfeng Huo:** software. **Jie Yao:** supervision. **Yongfei Bai:** formal analysis. **Xinyuan Hao:** funding acquisition. **Ying Zhang:** validation. **Shuqin Li:** data curation.

## Funding

This study was financially supported by the Center for Disease Control and Prevention of Shanxi Province through the second batch of “First‐Class” scientific research projects (05): “Evaluation of the toxicity of phillyrin and the study of its anti‐aging mechanism”.

## Conflicts of Interest

The authors declare no conflicts of interest.

## Supporting information


**Figure S1:** The treatment of mice and cells in each group. (A) The treatment of mice in each group. (B) The treatment of cells in each group.

## Data Availability

The data that support the findings of this study are available on request from the corresponding author. The data are not publicly available due to privacy or ethical restrictions.

## References

[fsn372241-bib-0001] Alzheimer's Association . 2021. “Alzheimer's Disease Facts and Figures.” Alzheimer's & Dementia 17, no. 3: 327–406. 10.1002/alz.12328 33756057

[fsn372241-bib-0002] Bai, Y. , J. Li , W. Liu , et al. 2015. “Pharmacokinetic of 5 Components After Oral Administration of Fructus Forsythiae by HPLC‐MS/MS and the Effects of Harvest Time and Administration Times.” Journal of Chromatography B, Analytical Technologies in the Biomedical and Life Sciences 993–994: 36–46. 10.1016/j.jchromb.2015.04.041 25984964

[fsn372241-bib-0003] Bettio, L. E. B. , L. Rajendran , and J. Gil‐Mohapel . 2017. “The Effects of Aging in the Hippocampus and Cognitive Decline.” Neuroscience and Biobehavioral Reviews 79: 66–86. 28476525 10.1016/j.neubiorev.2017.04.030

[fsn372241-bib-0004] Chen, E. , M. Li , Z. Liao , D. Yao , Y. Li , and L. Huang . 2024. “Phillyrin Reduces ROS Production to Alleviate the Progression of Intervertebral Disc Degeneration by Inhibiting NF‐κB Pathway.” Journal of Orthopaedic Surgery and Research 19, no. 1: 308. 38773639 10.1186/s13018-024-04695-y PMC11110443

[fsn372241-bib-0005] Chen, P. , Z. Guo , J. Lei , and Y. Wang . 2024. “Pomegranate Polyphenol Punicalin Ameliorates Lipopolysaccharide‐Induced Memory Impairment, Behavioral Disorders, Oxidative Stress, and Neuroinflammation via Inhibition of TLR4‐NF‐кB Pathway.” Phytotherapy Research 38, no. 7: 3489–3508. 10.1002/ptr.8219 38695373

[fsn372241-bib-0006] Cho, H. E. , S. Y. Ahn , I. S. Son , G. H. Hwang , and S. C. Kim . 2010. “HPLC‐Tandem Mass Spectrometric Analysis of the Marker Compounds in Forsythiae Fructus and Multivariate Analysis.” Journal of Pharmaceutical and Biomedical Analysis 53, no. 3: 478–483.

[fsn372241-bib-0007] Chung, H. Y. , M. Cesari , S. Anton , et al. 2009. “Molecular Inflammation: Underpinnings of Aging and Age‐Related Diseases.” Ageing Research Reviews 8, no. 1: 18–30. 18692159 10.1016/j.arr.2008.07.002 PMC3782993

[fsn372241-bib-0008] Coyle, J. T. , and P. Puttfarcken . 1993. “Oxidative Stress, Glutamate, and Neurodegenerative Disorders.” Science 262, no. 5134: 689–695. 7901908 10.1126/science.7901908

[fsn372241-bib-0009] Cui, W. , C. Sun , Y. Ma , S. Wang , X. Wang , and Y. Zhang . 2020. “Inhibition of TLR4 Induces M2 Microglial Polarization and Provides Neuroprotection via the NLRP3 Inflammasome in Alzheimer's Disease.” Frontiers in Neuroscience 14: 444. 32508567 10.3389/fnins.2020.00444 PMC7251077

[fsn372241-bib-0010] Cui, X. , P. Zuo , Q. Zhang , et al. 2006. “Chronic Systemic D‐Galactose Exposure Induces Memory Loss, Neurodegeneration, and Oxidative Damage in Mice: Protective Effects of R‐Alpha‐Lipoic Acid.” Journal of Neuroscience Research 83, no. 8: 1584–1590. 16555301 10.1002/jnr.20845

[fsn372241-bib-0011] Du, Y. , L. You , B. Ni , et al. 2020. “Phillyrin Mitigates Apoptosis and Oxidative Stress in Hydrogen Peroxide‐Treated RPE Cells Through Activation of the Nrf2 Signaling Pathway.” Oxidative Medicine and Cellular Longevity 2020: 2684672. 33101585 10.1155/2020/2684672 PMC7576358

[fsn372241-bib-0012] El‐Mesery, M. , A. El‐Mesery , O. Ahmed‐Farid , N. M. Abd El‐Aziz , and A. E. Abdel Moneim . 2024. “Geraniol Attenuates Oxidative Stress and Neuroinflammation‐Mediated Cognitive Impairment in D‐Galactose‐Induced Mouse Aging Model.” Naunyn‐Schmiedeberg's Archives of Pharmacology 397, no. 6: 4651–4665.

[fsn372241-bib-0013] Fang, X. , Y. Wang , J. Wang , J. Zhang , and X. Wang . 2013. “Microwave‐Assisted Extraction Followed by RP‐HPLC for the Simultaneous Extraction and Determination of Forsythiaside A, Rutin, and Phillyrin in the Fruits of *Forsythia suspensa* .” Journal of Separation Science 36, no. 16: 2672–2679. 23913652 10.1002/jssc.201300317

[fsn372241-bib-0014] Feng, Y. , Y. Cui , J. L. Gao , et al. 2016. “Resveratrol Attenuates Neuronal Autophagy and Inflammatory Injury by Inhibiting the TLR4/NF‐κB Signaling Pathway in Experimental Traumatic Brain Injury.” International Journal of Molecular Medicine 37, no. 4: 921–930. 26936125 10.3892/ijmm.2016.2495 PMC4790669

[fsn372241-bib-0015] Ganesan, B. , T. Gowda , A. Al‐Jumaily , K. N. K. Fong , S. K. Meena , and R. K. Y. Tong . 2019. “Ambient Assisted Living Technologies for Older Adults With Cognitive and Physical Impairments: A Review.” European Review for Medical and Pharmacological Sciences 23, no. 23: 10470–10481. 31841201 10.26355/eurrev_201912_19686

[fsn372241-bib-0016] GBD 2019 Dementia Forecasting Collaborators . 2022. “Estimation of the Global Prevalence of Dementia in 2019 and Forecasted Prevalence in 2050: An Analysis for the Global Burden of Disease Study 2019.” Lancet Public Health 7, no. 2: e105–e125. 34998485 10.1016/S2468-2667(21)00249-8 PMC8810394

[fsn372241-bib-0017] Jiang, Q. , J. Chen , X. Long , et al. 2020. “Phillyrin Protects Mice From Traumatic Brain Injury by Inhibiting the Inflammation of Microglia via PPARγ Signaling Pathway.” International Immunopharmacology 79: 106083. 31923823 10.1016/j.intimp.2019.106083

[fsn372241-bib-0018] Jiang, Q. , D. Wei , X. He , C. Gan , X. Long , and H. Zhang . 2021. “Phillyrin Prevents Neuroinflammation‐Induced Blood‐Brain Barrier Damage Following Traumatic Brain Injury via Altering Microglial Polarization.” Frontiers in Pharmacology 12: 719823. 34744713 10.3389/fphar.2021.719823 PMC8565465

[fsn372241-bib-0019] Li, X. , L. Wang , C. Li , et al. 2023. “Phillyrin Ameliorates Oxidative Stress in D‐Galactose‐Induced Senescence in the Brain of Mice by Regulating the Nrf2.” 18, no. 5: 1934578X231173237.

[fsn372241-bib-0020] Ma, J. , Z. Wang , Y. Sun , et al. 2024. “Phillyrin: A Potential Therapeutic Agent for Osteoarthritis via Modulation of NF‐κB and Nrf2 Signaling Pathways.” International Immunopharmacology 141: 112960. 39159565 10.1016/j.intimp.2024.112960

[fsn372241-bib-0021] Mattson, M. P. , S. L. Chan , and W. Duan . 2002. “Modification of Brain Aging and Neurodegenerative Disorders by Genes, Diet, and Behavior.” Physiological Reviews 82, no. 3: 637–672. 12087131 10.1152/physrev.00004.2002

[fsn372241-bib-0022] Moqri, M. , C. Herzog , J. R. Poganik , et al. 2023. “Biomarkers of Aging for the Identification and Evaluation of Longevity Interventions.” Cell 186, no. 18: 3758–3775. 37657418 10.1016/j.cell.2023.08.003 PMC11088934

[fsn372241-bib-0023] Omidi, M. , A. Ahangarpour , S. A. Mard , and L. Khorsandi . 2019. “The Effects of Myricitrin and Vitamin E Against Reproductive Changes Induced by D‐Galactose as an Aging Model in Female Mice.” International Journal of Reproductive Biomedicine 17, no. 11: 789–798. 31911961 10.18502/ijrm.v17i10.5486 PMC6906854

[fsn372241-bib-0024] Qi, L. F. , Y. Liu , S. Liu , et al. 2024. “Phillyrin Promotes Autophagosome Formation in A53T‐αSyn‐Induced Parkinson's Disease Model via Modulation of REEP1.” Phytomedicine 134: 155952. 39178680 10.1016/j.phymed.2024.155952

[fsn372241-bib-0025] Qu, X. Y. , Q. J. Li , H. M. Zhang , et al. 2016. “Protective Effects of Phillyrin Against Influenza A Virus in Vivo.” Archives of Pharmacal Research 39, no. 7: 998–1005. 27323762 10.1007/s12272-016-0775-z

[fsn372241-bib-0026] Rufini, A. , P. Tucci , I. Celardo , and G. Melino . 2013. “Senescence and Aging: The Critical Roles of p53.” Oncogene 32, no. 43: 5129–5143. 23416979 10.1038/onc.2012.640

[fsn372241-bib-0027] Saleh, D. O. , D. F. Mansour , I. M. Hashad , and R. M. Bakeer . 2019. “Effects of Sulforaphane on D‐Galactose‐Induced Liver Aging in Rats: Role of Keap‐1/Nrf‐2 Pathway.” European Journal of Pharmacology 855: 40–49. 31039346 10.1016/j.ejphar.2019.04.043

[fsn372241-bib-0028] Song, X. , M. Bao , D. Li , and Y. M. Li . 1999. “Advanced Glycation in D‐Galactose Induced Mouse Aging Model.” Mechanisms of Ageing and Development 108, no. 3: 239–251. 10405984 10.1016/s0047-6374(99)00022-6

[fsn372241-bib-0029] Sun, K. , P. Yang , R. Zhao , Y. Bai , and Z. Guo . 2018. “Matrine Attenuates D‐Galactose‐Induced Aging‐Related Behavior in Mice via Inhibition of Cellular Senescence and Oxidative Stress.” Oxidative Medicine and Cellular Longevity 2018: 7108604. 30598725 10.1155/2018/7108604 PMC6288577

[fsn372241-bib-0030] Teleanu, D. M. , A. G. Niculescu , I. I. Lungu , et al. 2022. “An Overview of Oxidative Stress, Neuroinflammation, and Neurodegenerative Diseases.” International Journal of Molecular Sciences 23, no. 11: 5938. 35682615 10.3390/ijms23115938 PMC9180653

[fsn372241-bib-0031] Wang, D. H. , X. He , and Q. He . 2019. “Combining Use of Phillyrin and Autophagy Blocker Alleviates Laryngeal Squamous Cell Carcinoma via AMPK/mTOR/p70S6K Signaling.” Bioscience Reports 39, no. 6: BSR20190459. 31147451 10.1042/BSR20190459 PMC6616054

[fsn372241-bib-0032] Wang, Z. , Q. Xia , X. Liu , et al. 2018. “Phytochemistry, Pharmacology, Quality Control and Future Research of *Forsythia suspensa* (Thunb.) Vahl: A Review.” Journal of Ethnopharmacology 210: 318–339. 28887216 10.1016/j.jep.2017.08.040

[fsn372241-bib-0033] Xia, Z. , M. Gao , P. Sheng , et al. 2022. “Fe(3)O(4) Nanozymes Improve Neuroblast Differentiation and Blood‐Brain Barrier Integrity of the Hippocampal Dentate Gyrus in D‐Galactose‐Induced Aged Mice.” International Journal of Molecular Sciences 23, no. 12: 6463. 35742908 10.3390/ijms23126463 PMC9224281

[fsn372241-bib-0034] Yan, L. Y. , M. J. Liu , H. R. Yan , X. Li , J. H. Xu , and J. X. Yang . 2015. “Study on Anti‐Aging Effects of Phillyrin on Aging Model Mice.” China Pharmacy 26, no. 1: 37–39.

[fsn372241-bib-0035] Zhang, D. , B. Qi , D. Li , et al. 2020. “Phillyrin Relieves Lipopolysaccharide‐Induced AKI by Protecting Against Glycocalyx Damage and Inhibiting Inflammatory Responses.” Inflammation 43, no. 2: 540–551. 31832909 10.1007/s10753-019-01136-5 PMC7095384

[fsn372241-bib-0036] Zhang, G. , and S. Ghosh . 2001. “Toll‐Like Receptor‐Mediated NF‐kappaB Activation: A Phylogenetically Conserved Paradigm in Innate Immunity.” Journal of Clinical Investigation 107, no. 1: 13–19. 11134172 10.1172/JCI11837 PMC198554

[fsn372241-bib-0037] Zhou, S. , A. Zhang , and W. Chu . 2019. “Phillyrin Is an Effective Inhibitor of Quorum Sensing With Potential as an Anti‐ *Pseudomonas aeruginosa* Infection Therapy.” Journal of Veterinary Medical Science 81, no. 3: 473–479. 30686799 10.1292/jvms.18-0523 PMC6451918

