# Supplementary material for: Phillyrin Alleviates Memory Impairment in D‐Galactose‐Induced Aged Mice by Suppressing Hippocampal Neuroinflammation via the TLR4/NF‐κB Signaling Pathway
Source: Food Sci Nutr. 2026 Aug 7;14(8):e72241. doi: 10.1002/fsn3.72241 (PMC13451633; doi:10.1002/fsn3.72241)
Supplement: Supplementary file 1 [file FSN3-14-e72241-s001.docx]

**Supplementary Figure1**

**Fig. S1.** The treatment of mice and cells in each group. (A) The treatment of mice in each group. (B) The treatment of cells in each group.
